# Partner randomized controlled trial: study protocol and coaching intervention

**DOI:** 10.1186/1471-2431-12-42

**Published:** 2012-04-02

**Authors:** Jane M Garbutt, Gabrielle Highstein, Yan Yan, Robert C Strunk

**Affiliations:** 1 Department of Pediatrics, Division of Allergy and Pulmonary Medicine, Washington University, St Louis, USA; 2 Department of Medicine, Washington University, St Louis, USA; 3 Winds of Change at Crosswinds, 154 Hatchville Road, East Falmouth, MA 02536, USA; 4 Department of Surgery, Washington University, St Louis, USA; 5 General Medical Sciences, Washington University School of Medicine, Campus Box 8005, 660S. Euclid Ave., St. Louis, MO 63110, USA

**Keywords:** Asthma, Behavioral skills training, Lay coaching

## Abstract

**Background:**

Many children with asthma live with frequent symptoms and activity limitations, and visits for urgent care are common. Many pediatricians do not regularly meet with families to monitor asthma control, identify concerns or problems with management, or provide self-management education. Effective interventions to improve asthma care such as small group training and care redesign have been difficult to disseminate into office practice.

**Methods and design:**

This paper describes the protocol for a randomized controlled trial (RCT) to evaluate a 12-month telephone-coaching program designed to support primary care management of children with persistent asthma and subsequently to improve asthma control and disease-related quality of life and reduce urgent care events for asthma care. Randomization occurred at the practice level with eligible families within a practice having access to the coaching program or to usual care. The coaching intervention was based on the transtheoretical model of behavior change. Targeted behaviors included 1) effective use of controller medications, 2) effective use of rescue medications and 3) monitoring to ensure optimal control. Trained lay coaches provided parents with education and support for asthma care, tailoring the information provided and frequency of contact to the parent's readiness to change their child's day-to-day asthma management. Coaching calls varied in frequency from weekly to monthly. For each participating family, follow-up measurements were obtained at 12- and 24-months after enrollment in the study during a telephone interview.

The primary outcomes were the mean change in 1) the child's asthma control score, 2) the parent's quality of life score, and 3) the number of urgent care events assessed at 12 and 24 months. Secondary outcomes reflected adherence to guideline recommendations by the primary care pediatricians and included the proportion of children prescribed controller medications, having maintenance care visits at least twice a year, and an asthma action plan. Cost-effectiveness of the intervention was also measured.

**Discussion:**

Twenty-two practices (66 physicians) were randomized (11 per treatment group), and 950 families with a child 3-12 years old with persistent asthma were enrolled. A description of the coaching intervention is presented.

**Trial registration:**

ClinicalTrials.gov identifier NCT00860834.

## Background

Asthma is the most common chronic disease of childhood, and in 2009 affected an estimated 7.1 million children in the United States (9.6%) [1]. Despite advances in preventive treatments, asthma morbidity is significant. In 2008, children with asthma missed 10.5 million school days, experienced 6.7 million office visits, 640,000 Emergency Department (ED) visits, 157,000 hospital admissions, and 185 died [1]. Each year, the direct costs for asthma in the United States are more than $8.1 billion, and in-patient hospital costs are over $3.5 billion [2]. Reducing asthma morbidity is a national healthcare objective [3]

In 1991, the National Asthma Education and Prevention Program (NAEPP) of the National Heart, Lung and Blood Institute (NHLBI) developed Guidelines for the Diagnosis and Management of Asthma,[4] with updates in 1997,[5] 2002,[6] and 2007 [7]. Many studies have demonstrated significant gaps between guideline recommendations and actual practice. Daily treatment with inhaled corticosteroids (ICS) or leukotriene receptor antagonists (LTRAs) is recommended to prevent asthma symptoms, activity limitations and minimize acute exacerbations, [8-16] but these effective controller medications are underused [17-20]. Recent physician surveys and pharmacy data suggest only 50-90% of eligible children are prescribed an effective controller medication, [20-28] and up to a third of parents do not fill their child's prescription [20,29]. When prescriptions are filled, adherence is poor [20,30-32]. Parents report that not knowing how to use asthma medications effectively, not believing them to be necessary--often due to conflicting advice from friends and relatives,[33] and concerns about efficacy, risks of long-term usage, cost and social stigmatization associated with inhaler use are significant barriers to regular use of controller medications [18,34-37].

Short-acting beta_2_-agonists are the treatment of choice for relieving acute symptoms,[5] and systemic corticosteroids may speed recovery and prevent recurrence of exacerbations [38,39]. Early treatment with these "rescue medications" in an acute exacerbation can reduce ED visits, improve patient outcomes, and can be managed effectively by the parent guided by a written Asthma Action Plan (AAP) [40]. Yet, many families come to the ED because of a delayed response to the child's early asthma signs, or failure to implement the steps detailed on their AAP [41-43].

Morbidity is reduced and ICS use is higher in patients who report regularly scheduled asthma visits [44,45]. The guidelines recommend 1) periodic assessments (every 1 to 6 months) to monitor asthma control, assess if the goals of therapy are being met, review medication use and the child's AAP, and adjust treatment as needed; 2) asthma self-management education at diagnosis, with review and reinforcement at every opportunity; 3) a partnership between the primary care provider (PCP), the patient and their family to develop shared treatment goals, select an appropriate treatment regimen, resolve asthma-related concerns, and provide support for day-to-day care. However, maintenance asthma care delivered by PCPs is not optimal [46,47]. Only 50% of asthmatic children report maintenance care visits twice a year,[48] 70% report some asthma education, and 30-50% have an AAP [1,46]. Pediatricians report that lack of familiarity with the complex guidelines, lack of agreement with some recommendations, low self-efficacy in their ability to improve patient's self-management behaviors, low expectation of improvements, and the perceived associated high workload deter them from following guideline recommendations [49-53]. In addition, logistical issues such as lack of time, educational materials, support staff, and inadequate reimbursement are significant barriers to guideline implementation [46,49,50,52].

Effective interventions to improve asthma care have been difficult to disseminate into office practice; many physicians are unwilling or unable to attend training sessions to improve their skills,[52,54-56] and most offices do not have a nurse or health educator available to share the work of asthma care or provide home visits [24,57-59]. Most efforts to redesign care delivery have been implemented in managed care settings, federally funded or specialist clinics and have limited potential to be translated into office-based care. Programs report improved quality of life, reduced school and work absences, and reduced ED visits and hospitalizations among patients who complete the program,[58,59] but may fail to reach up to two thirds of the target population [57,59].

Consumer demand can change physician behavior. Patients who ask for treatment are more likely to receive it than those who do not make a request [60]. Direct-to-consumer advertising has been shown to cause patients to seek more information about a product from their physicians and stimulate discussion about treatment options [61]. In addition, patients with chronic illnesses who actively participate in planning their treatment (ask questions, review treatment options, state preferences) are more likely to follow through with their treatment plan, and have better health outcomes than those who do not participate [62-66]. Participatory decision-making also increases patient satisfaction and retention [67].

Self-management education is most effective when it includes self management training and support and active sustained follow-up [68]. These are some of the key features of what we have called coaching--tailored education and support to improve asthma self-management delivered by lay health workers [69-71]. Lay or community health workers are from or like the community they serve in a relevant way, and have been used successfully to increase access to appropriate health care services for underserved minority populations [69,72,73].

In the PARTNER study (Parents, Pediatricians and Telephone Coaches Partner to Improve Control of Asthma), coaching is integrated into office-based asthma care without placing unrealistic expectations on the PCP or their staff. The lay asthma coach encourages parents to maintain active partnership with the pediatrician to improve asthma control and planning for risk events and provides tailored education about self-management behaviors. We hypothesized that integrating this pragmatic intervention into primary care would improve asthma outcomes for the child and their family and also improve asthma care provided by their PCP. The PARTNER Study tested this hypothesis in a randomized controlled trial (RCT) comparing outcomes for practices with access to the 12-month telephone coaching intervention for their patients to those from practices who provide usual care. Participant follow-up occurs 12 and 24-months after randomization. This paper describes the study protocol and the coaching intervention.

## Methods and design

### Design and setting

The coaching intervention is being evaluated in a stratified cluster randomized design, stratifying on office location (urban or suburban) and randomizing at the practice level, thus avoiding contamination within practices that would occur with randomization at the patient level. Study outcomes are measured at the level of the patient using a cohort design in which the same subjects from each PCP are tracked throughout the study. Practices were randomly assigned to the intervention or the control condition with all participating physicians within the same practice assigned to the same study group. Using billing data, each practice has identified potentially eligible families with a child who had received asthma care (ICD-9 493.XX). The study team contacted these families to invite participation, informing them of the study group assignment of their practice prior to obtaining their consent. Participants are followed for two years with outcomes measured by parent interviews and chart audit. The study was approved by Washington University Institutional Review Board and was conducted in 22 primary care pediatric practices in the Midwest.

### Participants

#### Practices and physicians

Practices were eligible to participate if they were community-based, affiliated with St Louis Children's Hospital and provided primary care for children, including asthma care for at least 40 children aged 3 to 12 years old. At each practice, physicians were eligible to participate in the study if they spent at least 50% of their time in general pediatrics and they were not an asthma specialist (allergist or pulmonary specialist).

#### Families

At each participating practice, families were eligible to participate in the trial if their child was 3 to 12 years old, and reported a physician diagnosis of asthma, and  ≥ 1 acute exacerbation within the prior year that required an unscheduled office visit, a course of oral steroids, an Emergency Department (ED) visit or hospitalization. Eligibility also required the parent to report that the child was using daily controller medications or had asthma symptoms consistent with persistent asthma for the past 2 weeks [5]. Families were excluded if they did not have a phone or could not speak English, if a sibling was already a study subject, or if the child was participating in another asthma study.

### Recruitment and consent

#### Practices and physicians

All potentially eligible pediatricians received a written invitation to participate and faxed back a form indicating their level of interest in study participation. The Principal Investigator (PI) met with each PCP who indicated their willingness to take part in order to explain the requirements for study participation. One pediatrician in each practice provided written consent. Although participation by all pediatricians in the practice was encouraged, it was not required. Each participating physician received access to the Education in Quality Improvement in Pediatric Practice (EQIPP) asthma module offered by the American Academy of Pediatrics, and each practice was paid $100 to compensate for administrative time required for study tasks.

#### Families

Each practice used billing data to provide a list of potentially eligible families to the study team. These families included those with a child between 3 and 12 years old who had received asthma care in the past year either at the office, in the Emergency Department (ED) or through the After Hours Call Center. Each physician was provided an opportunity to review their list of names to identify families they considered to be unsuitable for study participation.

All potentially eligible families received an invitation to participate in the study from their PCP and the study team. The mailed invitation included a business reply postcard and phone contact information to use to indicate their willingness to take part. If no reply was received within 2 weeks, a research assistant (RA) telephoned to repeat the study invitation, provide information about participation requirements and answer questions, and complete the eligibility questionnaire. Non-respondents received up to three phone contacts.

Consent forms were mailed to each eligible family who was interested in study participation. One parent (or legal guardian) per family provided written consent. Each family was paid $20 to $25 for completion of each of three study interviews. Parents who completed the consent and the baseline interview were enrolled in the study. Recruitment continued until all names provided by the practice had been processed, or the target for that practice was reached. The recruitment goal was an average of 40 families/practice.

#### The intervention and control conditions

### The coaching intervention

#### The intervention comprised

##### Interactions with PCPs

Each PCP in the intervention group had two 45-minute face-to-face meetings with the PIs (an asthma expert and the director of the local practice-based research network, PBRN). The goals of these meetings were to discuss the coaching program, determine a communication plan between the coach and the PCP, and address any issues with implementation. Intermittently during the study period asthma newsletters were circulated to all PCPs in the intervention group to provide updates about asthma treatment and address common issues in care identified by the coaches.

##### The coaching program

##### Program content

The program is based on the transtheoretical model of behavior change developed by James Prochaska [74-77]. Core constructs of the model are the Stages of Change, a series of five categories falling along a continuum of readiness (Precontemplation, Contemplation, Preparation, Action and Maintenance) to change a problem behavior. Movement forward and backward along the behavior change continuum is influenced by: the Pros and Cons for the desired behavior change; Self-efficacy or confidence in ones ability to change; and Cognitive/Affective Processes such as gathering information, being a role model, being moved emotionally, one's self-image, and social norms; and Behavioral Processes such as making a commitment, substituting, rewarding, cues, and social support. The targeted behaviors include: 1) effective use of controller medications, 2) effective use of rescue medications, and 3) monitoring to ensure optimal control.

##### The coaches

Four full-time lay coaches were recruited from the local community through advertising in the offices of participating practices and through the University. Three are mothers of children with asthma and one has experience with the transtheoretical model. Training covered asthma pathophysiology, asthma management, the transtheoretical model, building rapport, reflective listening and communication skills. The coaches were trained to "stage" a parent for each of the targeted behaviors, and tailor the information they provided to that assessment. Training activities included small group discussions, demonstrations, videos, experiential learning in the community, and role play. The coaches also learned by reviewing materials to be shared with the parents and by developing an annotated list of useful websites. The initial training took 4 weeks, and on-going training continued with weekly review of taped calls and discussions about topics of interest. The coaches provided support for each other.

The coach helped the parent to identify which of the targeted asthma care behaviors they wanted to work on. Triage questions and a written study protocol guided care advice. An example of triage questions and a treatment protocol is provided in Table 1. The coach encouraged the parent to set a short-term goal and provide a confidence score (1 to 10) for goal attainment in order to facilitate successful progress.

**Table 1:** Effective use of prescription controller medication(s)

STAGING QUESTIONS	ANSWERS	STAGE
**Has your child been prescribed a controller medication (e.g., Pulmicort, Flovent or Singulair)? If yes, how often is it supposed to be given?**	No Yes	NA Continue questions

Tell me about yesterday. **What medication(s) did you give?** **What time(s)?** **How about the day before that?**		Go back as far as you need to get a sense of whether the meds are being given correctly or not

**Is the controller medication(s) used every day as directed? How often are doses missed? How many missed doses in the last week?**	If has been doing it correctly for more than 6 months	Maintenance (I STILL AM)

*Possible Questions to help answer above question* **Do you have controller medications at home now?** **If no, why?** **Have you ever tried to use them every day? How did your child respond?** **Why did you stop?** **Do you think that controller medications help the asthma?**	If doing it correctly at least for the last week	Action (I AM)
	
	If doing it some of the time and intending to do better	Preparation (I WILL)
	
	If not doing it but thinking about how to do it	Contemplation (I MAY)
	
	If not doing it because bogged down	Precontemplation Believer (I CAN'T)
	
	If not doing it because don't believe in giving children regular meds	Precontemplation Nonbeliever (I WON'T)

##### Program delivery

Newly enrolled subjects were assigned a coach by the coach manager and mailed a package of PARTNER study materials for use in the coaching intervention including parent educational materials, an example of an Asthma Action Plan (AAP), and a schedule of asthma education classes available in the community.

Parents were called by their assigned coach within one week of enrollment and invited to participate in the coaching program. The coach provided an overview of the program goals and content, asked the parent about the child's asthma history, asthma treatment and treatment goals, and assessed the child's level of asthma control using questions based on the NAEPP guidelines [7]. The parent was asked to accept a short-term goal to read a booklet about asthma included in their study materials and to provide a confidence score from 1 to 10 to indicate the likelihood this goal would be reached in the next week. In this way, the coach introduced study processes used during the 12-month intervention to facilitate behavior change. The parent provided their contact information and a phone number for two relatives or friends who could be contacted if necessary. The second coaching call occurred one week later when the coach further described the program and the targeted behaviors, determined if the parent had achieved their short-term goal, staged the parent for each behavior and helped them to identify which behavior to target for improvement. Subsequent calls occurred weekly to monthly with the goal of addressing all three targeted behaviors during the 12-month intervention. Coaching calls were scheduled at times convenient for the parent and the coach and occurred during office hours from Monday to Friday, with evening appointments available until 8 pm one night per week. If necessary, the parent could leave a telephone message or send an email or text message to their coach to reschedule a call appointment or request an additional call. The coach was available to the parent for one year, and as much as possible, each family worked with the same coach.

The coaches were housed in an offsite call center to allow taping of calls and ensure the research assistants (RAs) remained blinded to group assignment during measurement calls. A sample of recorded calls was reviewed by the coach manager (weekly) and/or the PI (bi-weekly) to ensure program fidelity and provide feedback about management strategies. Calls were also reviewed by the coaches (self and peer review) as a learning resource.

The program was implemented in a flexible manner as determined by the parents' needs, circumstances and preferences. If a parent was initially unwilling to receive coaching help, the coach expressed understanding of the competing demands on the parent's time, and contacted the parent again in a low-key, non-demanding way to "check in," repeating this until the parent decided they wanted help with their child's asthma care. Anyone who asked directly not to be called was removed from the coach's call list, but could initiate calls to the coach any time during their 12-month enrollment in the program. The coach referred the parent to the PCP to discuss problems, develop or modify an AAP, or for training in use of asthma medications or equipment. The coach also distributed educational and other materials (such as a symptom diary), and referred the parent to community classes for asthma education or other community resources as needed. If significant personal problems that impacted asthma care were identified, the coach encouraged the parent to seek help from their own PCP or other advisors, and provided contact numbers for community resources. A resource manual was developed to assist the coach with these referrals and the PIs were available for consultation as needed.

The primary mechanism for communication between the coach and the PCP was through the parent. In addition, with the parent's permission, the coach provided a one-page faxed summary after 6-months and at the end of the program describing the child's asthma care and history of asthma control assessed during coaching calls. If the coach had particular concerns or was aware that the parent planned to return to their PCP for an asthma care visit, they sent a "concerns" fax detailing any current problems and relevant information for the PCP, and the PCP was invited to notify the coach if there was a particular problem they wanted the coach to address. The coach used forms developed for the study to guide their interaction with the parent, and record their activities.

##### The control condition

At the beginning of the study, all PCPs (intervention and control groups) received a flow chart for recommended administration of albuterol for worsening symptoms and information to help them overcome known barriers to additional maintenance care visits such as scheduling and billing difficulties [78,79]. They were also provided access to the EQIPP asthma module.

##### Measurements

All patient outcomes were measured by telephone interview of the parent or child, or by chart audit.

##### Physician measurements

All PCPs who signed the consent completed a brief, self-administered baseline questionnaire concerning their own and their practice demographics and features of asthma care provided for their patients. At the 24-month follow-up, all participating PCPs complete a self-administered questionnaire detailing the asthma care they provide, how they assess asthma control, and their confidence about providing asthma care. PCPs who participate in the intervention group provide feedback about the coaching program.

##### Participant measurements

##### Interviews

Interviews are conducted at baseline and 12 months to assess the impact of the intervention and at 24 months to assess if changes were sustained. Each interview was conducted by phone by a trained research assistant (RA) blinded to study group assignment and takes 20-30 minutes to complete. The baseline interview was completed after the signed parental consent was received or, with verbal consent, during the initial recruitment call. The 12-month and 24-month interviews were conducted on the appropriate anniversary of enrollment into the study using (call windows of 12 to 18 months and 24 to 27 months). If the parent could not be reached (answering machine, busy signal, no answer), multiple calls were made at different times of the day, on different days. Alternative numbers were called to allow other contacts to notify the parent. If the number was disconnected, it was called the following day and then weekly during the call window to see if it had been reconnected.

##### Content

For each interview, questions focused on asthma management, asthma outcomes, interactions with the PCP, and costs. Demographic information was collected at baseline.

The parent reported all medications used for asthma in the past week and whether or not they had an up-to-date asthma action plan (AAP). The child (if ≥ 5 years old) completed the Asthma Control Questionnaire (ACQ) (with permission) [80-83] to indicate their level of asthma control. The ACQ was also completed by the parent. Disease-specific quality of life (QOL) was measured for the parent using the validated, interviewer-administered 13-item Pediatric Asthma Caregiver's Quality of Life Questionnaire (PACQLQ) (with permission) [84]. Additional questions were asked about the frequency of asthma symptoms in the past 2 weeks and the number of days missed from school and work in the past year. Asthma risk was measured as reported urgent care episodes (events that required treatment with oral steroids or aerosolized bronchodilators in ED or office, or hospitalization for each 12-month measurement period). If the child received multiple types of urgent care on the same day, the most extensive level of treatment received was counted (hospitalization > ED > office visit). Using data from an entire calendar year avoids bias due to seasonal changes in the disease. Three questions from the Medical Outcomes Study [85] were use to assess the PCP's participatory style and an instrument developed by Wu and colleagues [86] was used to assess parent perceptions of asthma including their expectations about their child's asthma symptoms and functioning, concerns about controller medications, and competing family priorities. Parents also reported their costs for asthma medications and visits, and provided income information.

##### Chart audits

Audits of PCP charts assess office visits for asthma care over 3 time periods: the 12 months prior to enrollment and the two 12 month periods following enrollment. Asthma visits that occurred in each 12-month measurement period are categorized using a non-overlapping classification scheme.

##### Primary and secondary outcomes

The primary outcomes are a reduction in asthma impairment measured as improvement in asthma control and in asthma-related quality of life, and a reduction in asthma risk measured as a decrease in urgent care events. Secondary outcomes reflect changes in physician behavior, namely increased adherence to guideline-recommended maintenance care activities measured as the proportion of patients who report daily use of controller medications, an AAP and regular maintenance care visits. Aggregates of patient-level data are used to estimate physician behavior, recognizing that external factors such as parent behaviors and logistical issues may influence these measures.

##### Analytic plan

All main data analyses adhere to the intention-to-treat principle, quantifing the intervention effect both at the level of the cluster (practice), and at the level of the individual patient. In addition, a multilevel modeling strategy is used to explore the possible interaction of cluster and individual level covariates in modifying the intervention effect. A similar approach is taken for primary and secondary outcomes. Confounding factors are controlled for both in the study design (by stratification) and data analyses (regression modeling). All inferential analyses use a 2-tailed alpha of .05 to determine statistical significance. The cost-effectiveness and cost-benefit of the coaching program is assessed from society's viewpoint and from the point of view of an insurance company or large self-insured employer with sensitivity analysis to estimate the robustness of the results.

##### Sample size determination

The sample size and power determination considered the three primary outcomes: the change in ACQ scores from baseline to 12-months, the change in asthma-related QOL for the parent from baseline to 12-months measured with the PACQLQ, [84] and the probability of having ≥ 2 urgent care events within the first 12-months of the study. The Bonferroni method was used to allocate the type-I error rate equally among these three outcomes and maintain the overall study type-I error at 5%. Data from two prior studies were used to estimate the sample size [16,70].

For asthma control, assuming the between-group difference in the change between the baseline and 12 month ACQ scores to be 0.5 [83] and the standard deviation (SD) of the change in ACQ score of 0.90 in each group, then 11 or 12 practices in each arm with an average of 40 subjects/practice provides power of 97% and 99% respectively. For the parents' quality of life, assuming a 12-month change in PACQLQ scores of 0.26 (SD 1.18) in the control group and 0.66 (SD 1.18) in the intervention group, and the ICC of change in scores of 0.021, then 11 or 12 practices in each arm with an average of 40 subjects/practice provides power of 86% and 90% respectively. For urgent care events, assuming an interclass correlation coefficient (ICC) of 0.034, and a probability of urgent care events in the control group of 30%, and 16% in the intervention group, then 11 or 12 practices in each arm with an average of 40 subjects/practice provides power of 81% and 85% respectively.

We originally elected to have 12 practices in each arm, with an average of 40 subjects/PCP to ensure sufficient power for uncertainty in ICC estimates. However, due to pragmatic concerns, the Data Safety Monitoring Board (DSMB) agreed with using only 11 practices in each arm given that one solo PCP who was randomized to the intervention arm had a protracted illness that precluded our ability to implement the intervention within the study timeline, and another solo practice allocated to the control group was unable to provide patient lists. In addition, our recruitment target of 40 families/practice was met.

##### Chart audits

Audits of PCP charts assess office visits for asthma care over 3 time periods: the 12 months prior to enrollment and the two 12 month periods following enrollment. Asthma visits that occurred in each 12-month measurement period are categorized using a non-overlapping classification scheme.

##### Primary and secondary outcomes

The primary outcomes are a reduction in asthma impairment measured as improvement in asthma control and in asthma-related quality of life, and a reduction in asthma risk measured as a decrease in urgent care events. Secondary outcomes reflect changes in physician behavior, namely increased adherence to guideline-recommended maintenance care activities measured as the proportion of patients who report daily use of controller medications, an AAP and regular maintenance care visits. Aggregates of patient-level data are used to estimate physician behavior, recognizing that external factors such as parent behaviors and logistical issues may influence these measures.

##### Analytic plan

All main data analyses adhere to the intention-to-treat principle, quantifing the intervention effect both at the level of the cluster (practice), and at the level of the individual patient. In addition, a multilevel modeling strategy is used to explore the possible interaction of cluster and individual level covariates in modifying the intervention effect. A similar approach is taken for primary and secondary outcomes. Confounding factors are controlled for both in the study design (by stratification) and data analyses (regression modeling). All inferential analyses use a 2-tailed alpha of .05 to determine statistical significance. The cost-effectiveness and cost-benefit of the coaching program is assessed from society's viewpoint and from the point of view of an insurance company or large self-insured employer with sensitivity analysis to estimate the robustness of the results.

##### Sample size determination

The sample size and power determination considered the three primary outcomes: the change in ACQ scores from baseline to 12-months, the change in asthma-related QOL for the parent from baseline to 12-months measured with the PACQLQ, [84] and the probability of having ≥ 2 urgent care events within the first 12-months of the study. The Bonferroni method was used to allocate the type-I error rate equally among these three outcomes and maintain the overall study type-I error at 5%. Data from two prior studies were used to estimate the sample size [16,70].

For asthma control, assuming the between-group difference in the change between the baseline and 12 month ACQ scores to be 0.5 [83] and the standard deviation (SD) of the change in ACQ score of 0.90 in each group, then 11 or 12 practices in each arm with an average of 40 subjects/practice provides power of 97% and 99% respectively. For the parents' quality of life, assuming a 12-month change in PACQLQ scores of 0.26 (SD 1.18) in the control group and 0.66 (SD 1.18) in the intervention group, and the ICC of change in scores of 0.021, then 11 or 12 practices in each arm with an average of 40 subjects/practice provides power of 86% and 90% respectively. For urgent care events, assuming an interclass correlation coefficient (ICC) of 0.034, and a probability of urgent care events in the control group of 30%, and 16% in the intervention group, then 11 or 12 practices in each arm with an average of 40 subjects/practice provides power of 81% and 85% respectively.

We originally elected to have 12 practices in each arm, with an average of 40 subjects/PCP to ensure sufficient power for uncertainty in ICC estimates. However, due to pragmatic concerns, the Data Safety Monitoring Board (DSMB) agreed with using only 11 practices in each arm given that one solo PCP who was randomized to the intervention arm had a protracted illness that precluded our ability to implement the intervention within the study timeline, and another solo practice allocated to the control group was unable to provide patient lists. In addition, our recruitment target of 40 families/practice was met.

## Discussion

The PARTNER study is evaluating a business model for dissemination of asthma coaching into office practice. Subject recruitment is completed and 463 families have been randomized to the coaching intervention. During implementation of the PARTNER intervention the coaching process and quality monitoring procedures have been refined, and a manual and training program developed. Comparison of the processes in PARTNER with a previous telephone asthma coaching intervention, in which the coaches were nurses and worked part-time [70], has been instructive. Based on our experience in these two studies, we would propose that a coaching intervention include the following features:

• The coach should have shared experiences with the parents being coached. In particular, the shared experience of caring for a child with asthma is helpful in building the coaching relationship and establishing the credibility of the coaches. Parents seem to be more willing to engage with a peer coach than a nurse coach.

• The coach should be a full-time position. Each coach works 40 hours/week including one evening until 8 pm, with no weekend calls and works the same hours each week. Defined off time is important. Flexibility of work hours has made the position available to mothers of young children. Compared to a part-time model, full time employment of the coach provides easy access for training and support, and more opportunity for the coach to book and complete parent calls.

• Structured work hours, recurring appointments and a protocol to guide outreach efforts for parents who had missed an appointment facilitates successful contacts and use of additional communication channels (email, text messages) helps to schedule calls. Some parents are more willing to talk to the coach using cell phones rather than a landline, likely because they can verify the identity of the caller more easily via callback or text messaging to the coach's cellphone.

• Increasing the intensity of the intervention by increasing the number of calls during the 12-month intervention increases parents' willingness to actively participate in the coaching intervention. To date, the mean number of calls/family/year is 18.24 compared to the previous study where only 15% of participants had ≥ 9 calls.

• Ongoing training is essential: Coaches need to be knowledgeable about asthma, staging, and behavior change techniques, and have strong communication skills including reflective listening, empathy and telephone interviewing. The initial training included these skills, but ongoing monitoring and training is needed to maintain the quality of the intervention. Regular review of coaching calls (self-review, peer-review and review by managers) using a structured review tool together with written and oral feedback provides an essential learning experience for the coaches and the investigators.

• Locating the coaches in the same physical space fosters a collegial team environment and provides opportunity for social support and immediate feedback about a difficult call.

The coaching intervention may improve asthma control and disease-related quality of life and reduce urgent care events for asthma care. We hope that sharing the protocol and lessons learned will be helpful to other investigators interested in using a coaching intervention.

## Abbreviations

AAP: Asthma action plan; ACQ: Asthma control questionnaire; ED: Emergency department; EQIPP: Education in quality improvement in pediatric practice; DSMB: Data safety monitoring board; ICC: Intraclass correlation coefficient; ICS: Inhaled corticosteroids; LTRAs: Leukotriene receptor atagonists; NAEPP: National asthma education and prevention program; NHLBI: National heart lung: and blood institute; PACQLQ: Pediatric asthma caregiver's quality of life questionnaire; PARTNER: Parents pediatricians and telephone coaches partner to improve control of asthma; PCP: Primary care provider; PBRN: Practice-based research network; PI: Principal investigator; QOL: Quality of life; RA: Research assistant; RCT: Randomized controlled trial.

## Competing interests

The authors declare that they have no competing interests.

## Authors' contributions

JMG and RCS conceived the study and participated in it's design and coordination. JMG drafted the manuscript. GH designed the coaching intervention and trained the coaches. YY participated in the design of the study, did the sample size calculation, generated the random allocation sequence and assigned participants to interventions. All authors read and approved the final manuscript.

## Pre-publication history

The pre-publication history for this paper can be accessed here:

http://www.biomedcentral.com/1471-2431/12/42/prepub
